# SLoN: a spiking looming perception network exploiting neural encoding and processing in ON/OFF channels

**DOI:** 10.3389/fnins.2024.1291053

**Published:** 2024-03-06

**Authors:** Zhifeng Dai, Qinbing Fu, Jigen Peng, Haiyang Li

**Affiliations:** Machine Life and Intelligence Research Centre, School of Mathematics and Information Science, Guangzhou University, Guangzhou, China

**Keywords:** spiking looming perception network, ON/OFF channels, phase coding, eccentric down-sampling, looming selectivity

## Abstract

Looming perception, the ability to sense approaching objects, is crucial for the survival of humans and animals. After hundreds of millions of years of evolutionary development, biological entities have evolved efficient and robust looming perception visual systems. However, current artificial vision systems fall short of such capabilities. In this study, we propose a novel spiking neural network for looming perception that mimics biological vision to communicate motion information through action potentials or spikes, providing a more realistic approach than previous artificial neural networks based on sum-then-activate operations. The proposed spiking looming perception network (SLoN) comprises three core components. Neural encoding, known as phase coding, transforms video signals into spike trains, introducing the concept of phase delay to depict the spatial-temporal competition between phasic excitatory and inhibitory signals shaping looming selectivity. To align with biological substrates where visual signals are bifurcated into parallel ON/OFF channels encoding brightness increments and decrements separately to achieve specific selectivity to ON/OFF-contrast stimuli, we implement eccentric down-sampling at the entrance of ON/OFF channels, mimicking the foveal region of the mammalian receptive field with higher acuity to motion, computationally modeled with a leaky integrate-and-fire (LIF) neuronal network. The SLoN model is deliberately tested under various visual collision scenarios, ranging from synthetic to real-world stimuli. A notable achievement is that the SLoN selectively spikes for looming features concealed in visual streams against other categories of movements, including translating, receding, grating, and near misses, demonstrating robust selectivity in line with biological principles. Additionally, the efficacy of the ON/OFF channels, the phase coding with delay, and the eccentric visual processing are further investigated to demonstrate their effectiveness in looming perception. The cornerstone of this study rests upon showcasing a new paradigm for looming perception that is more biologically plausible in light of biological motion perception.

## 1 Introduction

Looming perception is an essential ability of sighted animals that detects objects moving in depth, accordingly plays critical roles in their daily movements including escaping from predators, preying, and so forth. With the development of robotic technologies, the vast majority of mobile robots nowadays are capable of detecting and avoiding collisions through obtaining sensor information from their surroundings, for example, infrared (Benet et al., 2002), ladar (Manduchi et al., 2005), and ultrasonic (Nelson and MacIver, 2006). Meanwhile, vision-based sensing modalities are benefiting from their economy, energy saving, and high-dimension feature acquisition, thus gradually prevailing over other collision sensing techniques in mobile robotics (Fu et al., 2019) and ground vehicles (Mukhtar et al., 2015). However, in terms of reliability and robustness in complex and dynamic environments, current approaches are far from acceptable for serving human society.

On the other hand, humans and animals possess highly robust and efficient dynamic vision systems to handle looming perception in fast-changing visual environments, which has always been inspiring researchers to explore the biological visual systems in order to develop robust artificial vision systems for addressing real world challenges. In this regard, early studies of mammalian vision began at the last century of 1930s, in which Hartline pointed out that the function of brightness increase (ON) and decrease (OFF) in early visual motion is separated into parallel processing pathways (Hartline, 1938). This means that the visual neural systems evolved to split motion information in parallel for better adapting to dynamic environments, with probably more energy consumption. After that, neuro-scientists studied the receptive fields (RF) of cats and rabbits and, in the 1960s, discovered direction-selective neurons that are sensitive to ON/OFF moving stimuli along specific directions (Hubel and Wiesel, 1962; Barlow and Levick, 1965). In the 1980s, Schiller et al. summarized the function of ON and OFF channels in the mammalian visual system (Schiller et al., 1986); he pointed out that ON/OFF retinal ganglion cells constitute the two polarity pathways of early visual processing.

With advances in biotechnology, the mammalian visual system has been better understood in recent decades. Many studies have demonstrated how the mammalian visual systems detect looming objects by recording the rats' reactions to the sight of an approaching disc (Yilmaz and Meister, 2013; Busse, 2018; Lee et al., 2020). In addition, scientists found that photoreceptors in the mammalian visual systems are not evenly distributed in the retina, with rods and cones having a high density in the foveal region and decreasing toward the marginal regions (Harvey and Dumoulin, 2011; Wurbs et al., 2013). At the same time, the size of individual RF increases toward the periphery. This results in a biological vision that images the foveal region clearly, while the peripheral region is relatively blurry. These visual characteristics of mammals have also inspired modeling works accounting for motion perception (Borst and Helmstaedter, 2015; Fu, 2023). However, the underlying circuits and mechanisms of biological vision remain largely unknown.

Although the biological substrates are elusive, computational modeling is particularly useful for testing hypotheses on biological signal processing. Regarding computational modeling of looming perception visual systems, there are many methods inspired by insects' visual systems (Fu et al., 2019). Fu et al. introduced a series of visual neural networks based on the locust's lobula giant movement detector-2 (LGMD2) neuronal mechanism, mimicking its distinctive looming selectivity for darker objects approaching relative to the background through bio-plausible ON/OFF pathways (Fu and Yue, 2015; Fu et al., 2018). Owing to the efficiency in hardware implementation, the model was applied to the vision systems of micro-robots for quick collision detection in navigation (Fu et al., 2016). More specifically, the object movement will elicit the image brightness to change with respect to time. When the brightness increases, the stimulus will enter the ON pathway, while the brightness decreases will enter the OFF pathway. The significance of ON/OFF channels has been systematically investigated through a recent research upon implementing different selectivity in motion perception (Fu, 2023). Moreover, there are also many other models for motion detection that employ the ON/OFF channels, such as the elementary motion detectors (EMD) (Franceschini, 2014). Franceschini et al., for the first time, proposed the splitting of EMD inputs into ON-EMD and OFF-EMD structures encoding light and dark moving edges separately for micro-simulation of photoreceptors in RF (Franceschini et al., 1989). Subsequently, following advancements in physiological research on the fruit fly *Drosophila*, various computational models employing different combinations of ON/OFF-EMD were proposed to simulate the motion vision of the fly (Eichner et al., 2011; Joesch et al., 2013; Fu and Yue, 2020). Additionally, models for small target motion detectors (STMDs) were developed, drawing inspiration from insects such as dragonflies and hoverflies. In these models, the ON/OFF channels play an indispensable role in discriminating between small moving targets and cluttered, dynamic backgrounds (Halupka et al., 2011; Wiederman et al., 2013).

However, these bio-inspired motion perception models essentially belong to the second generation of artificial neural networks (ANN), where neurons within the network transmit and calculate real numbers, which are weighted, summed, and then activated for delivery. Such approaches are not as realistic as biological visual systems that encode external stimuli as action potentials or spikes for signal communication between neurons. Specifically, when the pre-synaptic neuron receives a stimulus large enough, it is charged to transmit a spike to communicate with the post-synaptic neuron. Encoding spikes with neuronal dynamics concerning time gives rise to the third generation of spiking neural networks (SNNs) (Tan et al., 2020). In terms of energy efficiency, the SNN undoubtedly prevails over the previous generations of ANN as the SNN transmits binary information without multiplication before summation. In addition, sparse and asynchronous signal processing can also be allowed in SNN (Lobo et al., 2020). The vast majority of SNN modeling works have been proposed for addressing real-world challenges including object detection (Cordone et al., 2022), pattern recognition (Kheradpisheh et al., 2018; Tavanaei et al., 2019), and perceptual system (Masuta and Kubota, 2010; Tan et al., 2020). Recently, Yang et al. (2022, 2023) and Yang and Chen (2023a,b) offered new insights into enhancing spike-based machine learning performance using advanced information-theoretic learning methods. These studies have introduced novel frameworks to enhance SNN performance in specific tasks, allowing the model to achieve high-level intelligence, accuracy, robustness, and low power consumption compared with state-of-the-art artificial intelligence works.

In terms of motion perception, to the best of our knowledge, there are very few modeling works based on the SNN because decoding video signals to extract motion cues with temporal coherence is challenging. Salt (2016) and Salt et al. (2017, 2020) introduced a collision perception SNN implemented in an aerial robot. However, this study lacked systematic testing in a closed-loop flight scenario. Recently, the development of dynamic vision sensors (DVS) has significantly bolstered computer vision and vision-based robotic applications, e.g., Posch et al. (2014), Milde et al. (2015), Milde et al. (2017), Vasco et al. (2017), and Gallego et al. (2022). Compared with traditional, frame-based sensors, the DVS, such as, event-based camera features low latency, high speed, and high dynamic range. Most importantly, it can report ON (onset)/OFF (offset) motion events, which aligns much closer to the revealed principles of biological visual processing. Utilizing input from DVS, a novel spiking-EMD (sEMD) model was proposed for encoding ON/OFF motion events and decoding the direction of motion via the “time-of-travel” concept (Milde et al., 2018; D'Angelo et al., 2020). However, the cost of DVS is high, limiting its flexibility in mobile robotics due to its larger size and higher power requirements. Here, we aim to develop a more general method for SNN in motion perception that can be used with either frame-based or event-driven sensors.

Accordingly, this study introduces a novel SNN model, the Spiking Looming perception Network (SLoN), which offers two-fold advantages over previous ANN models. On one hand, the SLoN is more biologically realistic as it encodes visual streams into spikes for communication within the network. On the other hand, the SLoN mimics the receptive field (RF) of mammalian vision for looming perception with new bio-plausible concepts and mechanisms to achieve specific looming selectivity. The cornerstone of this study is based on the following aspects:

- In consonance with mammalian motion vision (Fu, 2023), the SLoN introduces ON/OFF channels that split the input signals into parallel pathways for neural encoding and down-sampling. This structure enables the SLoN to achieve varying looming selectivity to ON/OFF-contrast stimuli, improving robustness in complex and dynamic visual scenarios.
- Various neural coding models transform continuous signals into spatiotemporal spike trains, generally categorized as rate-based and temporal coding methods (Guo et al., 2021). Currently, neural coding is predominantly used to convert images into spike trains over a time window, with limited application in processing sequences of images for extracting motion information with temporal coherence. In the proposed SLoN model, phase coding is employed to convert video signals into spike trains as input. The phase coding method encodes video signals into spike trains using a binary representation, dividing each frame into eight periods with weighted spikes and emphasizing the significance of early spikes (Kim et al., 2018). Importantly, a new concept of phase delay (PD) is introduced to capture the spatiotemporal competition between excitatory and inhibitory currents within the ON/OFF channels, generating specific looming selectivity. The phase coding with delay mechanism not only combats noise but also portrays the fundamental characteristics of interactions between excitatory and inhibitory signals at a small time scale. This enables the proposed SNN model to process data from frame-based cameras.
- The mammalian receptive field (RF) does not sample as uniformly as traditional cameras (D'Angelo et al., 2020). To align with this, a strategy of eccentric down-sampling (EC) is adopted at the entry of ON/OFF channels of the proposed SLoN. The EC uses square regions to approximate the round retina of the animal, maintaining a quadrilateral camera resolution. Each RF spatially integrates information within its sensitive region through leaky integrate-and-fire (LIF) neuron models. The EC thus implements the foveal region with higher acuity to motion. Compared with previous study, this study delineates the EC for a better understanding of its computer implementation.

The rest of this study is structured as follows. Section 2 introduces the framework of SLoN with elaborated algorithms and illustrations, the setting of network parameters and experiments. Section 3 presents the systematic experimental results with analysis. Section 4 provides further discussions and concludes this research. The nomenclature used in this study is shown in Table 1.

**Table 1:** Nomenclature of this study.

**ANN**	**Artificial neural network**
DVS	Dynamic vision sensor
EC	Eccentric down-sampling
EPSC	Excitatory post-synaptic current
FFI	Feed-forward inhibition
IPSC	Inhibitory post-synaptic current
LGMD	Lobula giant movement detector
LIF	Leaky integrate-and-fire
PC	Phase coding
PD	Phase delay
RF	Receptive field
sEMD	Spiking elementary motion detector
SLoN	Spiking looming perception network
SNN	Spiking neural network

## 2 Materials and methods

**Figure 1:**
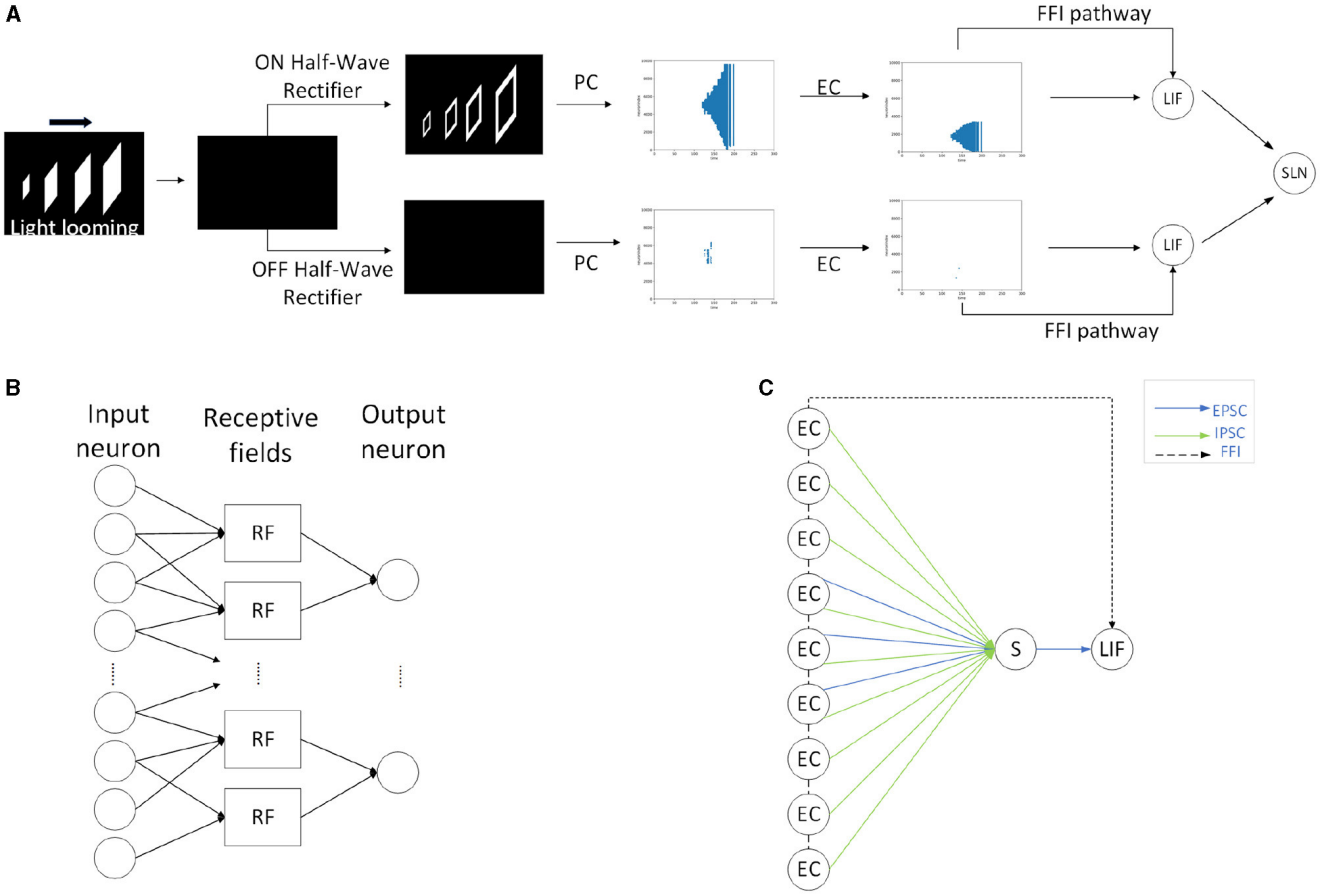
The complete structure of the spiking looming perception network (SLoN): **(A)** Exemplifies the signal processing diagram of the SLoN against a light looming square. The network captures differential images between successive frames and splits luminance change into ON/OFF channels by half-wave rectifiers. The motion information is then transformed into spike trains through phase coding and eccentric down-sampling. There are excitatory and inhibitory post-synaptic current (EPSC and IPSC) generated within ON/OFF channels. There is a feed-forward inhibition (FFI) pathway, and the ON/OFF-type LIF neurons convey polarity spikes to the output SLoN neuron. **(B)** Illustrates the diagram of the local LIF neuronal network structure for EC. **(C)** The Schematic diagram of the spiking layer representing transmission and computation of EPSC and IPSC, both aggregated at the summation (S) cell, then fed into the ON/OFF-type LIF neurons. The inhibition shares a larger scale than the excitation. If the population firing rate is greater than the threshold, EC cells will directly suppress the LIF cell through the FFI channel.

**Figure 2:**
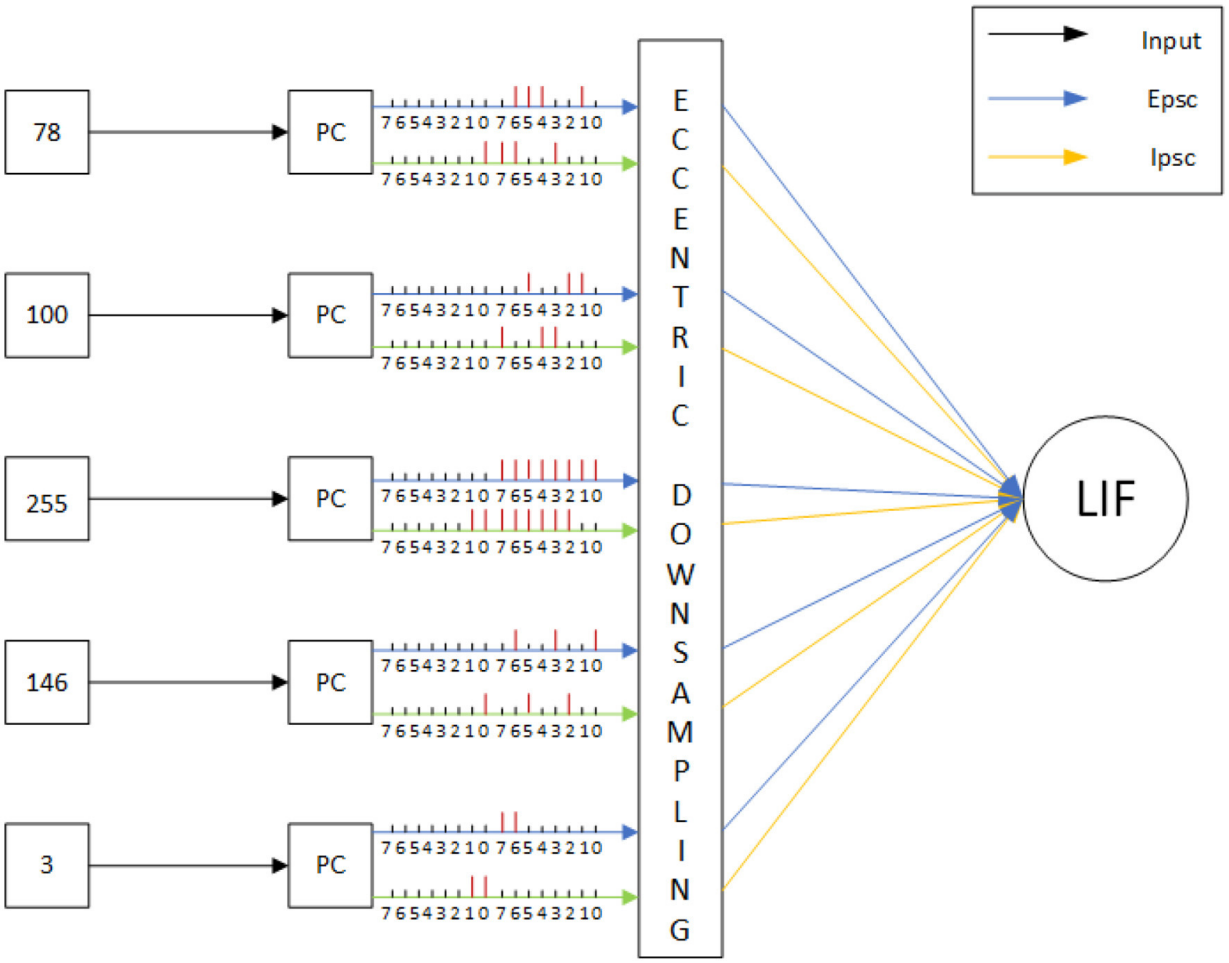
Illustration of the proposed phase coding with delay of IPSC: intensities of visual streams are transformed into spike trains with which each period consists of eight phases. Two periods herein are shown for each RF as the new concept of PD. Spikes are indicated as vertical red bars. In this figure, the delay is set at 2 phases.

**Figure 3:**
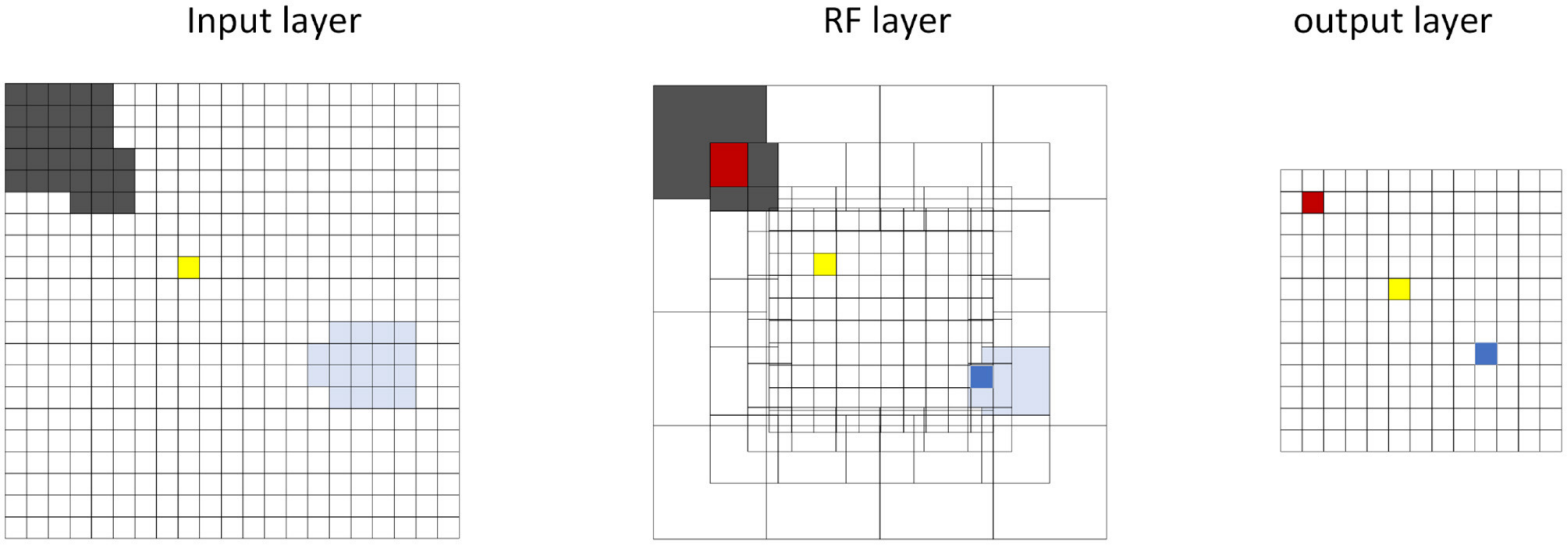
Schematic diagram of the eccentric down-sampling in the proposed SLoN: The neurons in the output layer are spatially distributed over the RF layer, taking the red, yellow, blue neurons as examples to illustrate how the RF of input layer has been down-sampled. The outside area of RF has larger intersects, whereas the inner area has one-to-one correspondence of RF (yellow neuron).

Within this section, we propose the methodology, including the framework of SLoN, the parameter setups, and the experimental settings. As the underlying mechanisms of biological looming perception visual systems remain largely elusive, this SNN model is inspired by a few relevant studies. These include the modeling of ON/OFF channels in the locust's LGMD model to separate different looming selectivities to ON/OFF-contrast stimuli (Fu et al., 2020a), eccentric down-sampling methods mimicking the mammalian receptive field (D'Angelo et al., 2020), and phase coding (Kim et al., 2018) with the inaugural concept of phase delay to process and correlate video signals. The complete structure of SLoN is shown in Figure 1. The EC and PD are shown in Figures 2, 3, respectively.

### 2.1 Framework of SLoN

In general, the proposed SLoN consists of three layers: the neural encoding layer, the spiking interaction layer, and the output layer. Specifically, the main difference from the vast majority of SNNs is that the SLoN prioritizes motion retrieval from videos to respond to looming motion features rather than handling singular images. Accordingly, within the first layer, the differential images between successive frames are split into ON/OFF channels encoding luminance increments/decrements, respectively. Phase coding is adopted to transform pixel intensity features into spatial-temporal spike trains, where each period is represented by eight phases. Importantly, the most significant information is retained in the first phases of each period, with the significance decaying by phase. The ON/OFF spike trains are then fed into an eccentric down-sampling mechanism, a partial neural network with LIF neurons, to simulate mammalian vision with higher acuity in the foveal region.

Within the second, spiking interaction layer, both EPSC and IPSC are generated within the ON/OFF channels. The inhibitory signals are temporally phase-delayed and spatially influenced by a larger field relative to the excitatory ones. This competition between them effectively responds most strongly to the expanding edges of objects over other types of motion stimuli. The ON/OFF-type LIF neurons integrate the corresponding EPSC and IPSC to generate polarity spikes. In addition, there are feed-forward inhibition (FFI) pathways that can directly suppress the ON/OFF-type LIF neurons if either the population rate exceeds a threshold. At the last layer, the output SLoN neuron integrates spikes from the ON/OFF channels, which inherently is another LIF neuron.

#### 2.1.1 ON/OFF motion retrieving

At the initial step, the SLoN does not handle every single image from a visual stream. Instead, motion information between two adjacent frames is retrieved. Assume *L*(*x, y, f*)∈*R*^3^ is the *f*th frame image brightness value, where *x*, *y* denote spatial positions, we can obtain the motion information as


(1) $$\begin{array}{l} P\left(x,y,f\right)=L\left(x,y,f\right)-L\left(x,y,f-1\right) \end{array}$$

As movement inevitably induces brightness increment and decrement within receptive field, the motion information is spit into parallel ON/OFF channels. This is achieved by half-wave rectifiers where the brightness increments/decrements flow into ON/OFF channels, respectively, mathematically expressed as


(2) $$\begin{array}{l} P_{on}\left(x,y,f\right)={\left[P\left(x,y,f\right)\right]}^{+}+\alpha_{1}P_{on}\left(x,y,f-1\right) \end{array}$$

(3) $$\begin{array}{l} P_{off}\left(x,y,f\right)={\left[P\left(x,y,f\right)\right]}^{-}+\alpha_{1}P_{off}\left(x,y,f-1\right) \end{array}$$

where α_1_ is a coefficient, which stands for the residual information in time. [*x*]^+^, [*x*]^−^ are denoted as *max*(*x*, 0) and *max*(−*x*, 0), respectively.

#### 2.1.2 Phase coding with weighted spikes

There are many neural coding methods as reviewed in the study by Guo et al. (2021), such as rate coding (Heeger, 2000), time-to-first-spike coding (Park et al., 2020), and burst coding (Eyherabide et al., 2009). Among these, phase coding was initiated in relation to the oscillatory firing pattern of neurons (O'Keefe and Recce, 1993; Laurent, 1996). Laurent conducted biological experiments on locusts, where a specific odor was directed toward one antenna of a locust. Laurent observed an oscillatory response in the mushroom body calyx of the locust. Building on this foundation, Kim et al. (2018) introduced an enhanced phase coding method with weighted spikes, emphasizing timing information. In this approach, the processing time of the stimulus is divided into k phases, and the stimulus is converted into binary values. Each phase is assigned a 0 or 1 (1 for a spike and 0 for no spike), with different weights assigned to each phase highlighting the importance of early information. The weight decays significantly concerning the phase time in each period, indicating that the most critical information is conveyed in the earlier phases. The proposed SLoN aligns with this coding strategy but introduces a novel concept of phase delay to accommodate spatial-temporal interactions between excitatory and inhibitory signals, aiming to shape the looming selectivity. Taking a few examples in Figure 2, the phase coding divides motion information, i.e., intensities of luminance change in ON/OFF channels into eight phases that constitute a time unit of computer processing. The weight of each phase is 2^−1^, 2^−2^, …, 2^−8^, and then the algorithm assigns 0, 1 to each phase with different weights. The computation can be defined as


(4) $$\begin{array}{l} C_{on}\left(x,y,8\left(f-1\right)+i\right)=mod\left(\frac{P_{on}\left(x,y,f\right)}{2^{i}},2\right),\;i=0,1,\ldots ,7 \end{array}$$

(5) $$\begin{array}{l} C_{off}\left(x,y,8\left(f-1\right)+i\right)=mod\left(\frac{P_{off}\left(x,y,f\right)}{2^{i}},2\right),\;i=0,1,\ldots ,7 \end{array}$$

where *mod*(*x, y*) is the modulus function that divides *x* by *y*. We herein use time phase instead of video frame to represent time t. *C*_*on*/*off*_(*x, y*, 8(*f*−1)+*i*) thus can be expressed as *C*_*on*/*off*_(*x, y, t*) in the following equations defined in this section. The ON/OFF-type spike trains can be obtained by assembling phasic spikes, spatially. Then, the weight of each phase in every period is computed as


(6) $$\begin{array}{l} \omega \left(t\right)=2^{-\left(1+mod\left(t,8\right)\right)}. \end{array}$$

#### 2.1.3 Eccentric down-sampling at the entrance of ON/OFF channels

Eccentric down-sampling uses a square RF to approximate a circular retina, as shown in Figure 3. The RF decreases linearly from the edge to the foveal area, where each RF corresponds to a single image pixel. First, we define the size and center of the RFs. The same sized RFs form a square ring. We can define only the position of upper left corner of each square ring, and then the foveal region will be defined. Accordingly, the size and center of the upper left corner of RFs can be computed as


(7) $$\begin{array}{l} R^{c}\left(i\right)=R^{c}\left(i-1\right)+\frac{1}{2}R^{s}\left(i-1\right),\;i=1,2,\ldots ,l \end{array}$$

(8) $$\begin{array}{l} R^{s}\left(i\right)=max\left(R^{s}\right)-\frac{max\left(R^{s}\right)}{d_{fovea}}R^{c}\left(i\right),\;i=1,2,\ldots ,l \end{array}$$

where *i* is the number of layers of the square ring, *R*^*s*^(*i*) represents the size of the RF of the upper left corner of the *i*^*th*^ layer, and *R*^*c*^(*i*) represents the center of the RF of the upper left corner of the *i*^*th*^ layer. *max*(*R*^*s*^) indicates the size of the outermost edge of the RF, and *d*_*fovea*_ is the total distance from the periphery to the edge of the foveal region whose dimension is 10% of the size of the image. *l* is the last layer number of edge square ring and *R*^*s*^(*l*+1) <2. The square ring starts at layer-0. The length of the upper left corner of the 0th layer square ring is *max*(*R*^*s*^), and its center is $\left(\frac{max\left(R^{s}\right)}{2},\frac{max\left(R^{s}\right)}{2}\right)$. For ease of description, we arrange all RFs in a column as a set A: *A* = {*RF*_1_, *RF*_2_, …, *RF*_*m*_}, where the side of *RF*_*k*_ is $R_{k}^{s}$ and its center position is $\left(R_{k}^{x},R_{k}^{y}\right)$. Basically, the EC herein is equivalent to a local SNN, and each RF is modeled as an LIF neuron (Gerstner et al., 2014), which integrates the information of the pixels it contains. When the membrane potential of the RF exceeds the threshold, it fires a spike to the next layer of its connected neurons. Taking the ON channel for example, the entire process can be defined mathematically as


(9) $$\begin{array}{l} M_{on}\left(k,t\right)=M_{on}\left(k,t_{1}\right)e^{-\frac{dt}{\tau }}+\overset{\left\lfloor \frac{R_{k}^{s}}{2}\right\rfloor }{\underset{i=\left\lfloor -\frac{R_{k}^{s}}{2}\right\rfloor }{\sum }}\overset{\left\lfloor \frac{R_{k}^{s}}{2}\right\rfloor }{\underset{j=\left\lfloor -\frac{R_{k}^{s}}{2}\right\rfloor }{\sum }}\frac{C_{on}\left(R_{k}^{x}+i,R_{k}^{y}+j,t\right)}{R_{k}} \end{array}$$

(10) $$S_{on}(k,t)=\{\begin{array}{ll} 1 & \text{ if }M_{on}(k,t)\geq \rho \\ 0 & \text{ if }M_{on}(k,t)<\rho \end{array}$$

where *M*_*on*_(*k, t*) represents the membrane potential of *RF*_*k*_, *t*_1_ indicates the moment of last emitted spike, and *R*_*k*_ is a percentage area of the RF. *R*_*k*_ affects the amount of current received by the pixel which *RF*_*k*_ contains. ⌊·⌋ denotes the floor function, and *S*_*on*_(*k, t*) indicates whether the *RF*_*k*_ emits spike at moment t. ρ is a spiking threshold for each *RF*_*k*_, and τ is a time constant ($\tau =\frac{1,000}{fps}$, fps represents frames per second, i.e., the sampling frequency). Next, we discuss which RFs the output neurons are connected to. We rezone the image, for which we need to take out a series of points. Denote the set of all upper left corner points of the square ring at the edge as a set B: *B* = {(*x*_0_, *y*_0_), (*x*_1_, *y*_1_), …, (*x*_*l*_, *y*_*l*_)}, the set of diagonal points in the foveal region as a set C: *C* = {(*x*_*l*+1_, *y*_*l*+1_), (*x*_*l*+2_, *y*_*l*+2_), …, (*x*_*l*+*n*_, *y*_*l*+*n*_)}, and the set of all bottom right corner points of the square ring at the edge as a set D: *D* = {(*x*_*l*+*n*+1_, *y*_*l*+*n*+1_), (*x*_*l*+*n*+2_, *y*_*l*+*n*+2_), …, (*x*_2*l*+*n*+1_, *y*_2*l*+*n*+1_)}, and the entire process can be defined mathematically as


(11) $$\begin{array}{l} x_{i}=y_{i}=R^{c}\left(i\right)-\frac{1}{2}R^{s}\left(i\right);\;i=1,\ldots ,l \end{array}$$

(12) $$\begin{array}{l} x_{l+1}=y_{l+1}=R^{c}\left(l\right),x_{l+i}=y_{l+i}=x_{l+i-1}+1;\;i=2,3,\ldots ,n \end{array}$$

(13) $$\begin{array}{l} x_{l+n+i}=y_{l+n+i}=L-x_{l-i+1};\;i=1,\ldots ,l+1 \end{array}$$

where *L* denotes the side length of the image, n is the dimension of the foveal region which starts at *R*^*c*^(*l*), and (*x*_0_, *y*_0_) is (0, 0). Now, we re-divide the image into regions and the delineated areas are


(14) $$\begin{array}{l} {△}_{n}^{\left(x\right)}=\left[x_{n},x_{n+1}\right],{△}_{n}^{\left(y\right)}=\left[y_{n},y_{n+1}\right];\;n=0,1,\ldots ,2l+n \end{array}$$

${△}_{n}^{\left(x\right)},{△}_{n}^{\left(y\right)}$ denote the *n*th region of *x*-axis and *y*-axis in Cartesian coordinate system, respectively. From this, we can establish the connection between the RF and the output neurons as


(15) $$\begin{array}{l} R\left(\tilde{x},\tilde{y}\right)=\left\{RF_{k}|\left(x,y\right)\cap \left({△}_{\tilde{x}}^{\left(x\right)}\times {△}_{\tilde{y}}^{\left(x\right)}\right)\neq \emptyset ,\;\left(x,y\right)\in RF_{k},\;k=1,2,\ldots ,m\right\} \end{array}$$

$R\left(\tilde{x},\tilde{y}\right)$ represents the RF which the output neuron $\left(\tilde{x},\tilde{y}\right)$ fastened. Furthermore, the output of neuron $\left(\tilde{x},\tilde{y}\right)$ can be calculated as


(16) $${\tilde{M}}_{on}(\tilde{x},\tilde{y},t)={\tilde{M}}_{on}(\tilde{x},\tilde{y},t_{2})e^{-\frac{dt}{\tau }}+\;\sum_{RF_{k}\in R(\tilde{x},\tilde{y})}S_{on}(k,t)$$

(17) $${\tilde{S}}_{on}(\tilde{x},\tilde{y},t)=\{\begin{array}{ll} 1 & \text{ if }\tilde{M}(\tilde{x},\tilde{y},t)\geq \rho \\ 0 & \text{ if }\tilde{M}(\tilde{x},\tilde{y},t)<\rho \end{array}$$

where *t*_2_ indicates the moment of last emitted spike, $\tilde{M_{on}}\left(\tilde{x},\tilde{y},t\right)$, ${\tilde{S}}_{on}\left(\tilde{x},\tilde{y},t\right)$ represents the membrane potential and spike of the neuron at $\left(\tilde{x},\tilde{y}\right)$. The eccentric down-sampling computations of OFF channels align with ON channels, which is not explicitly reiterated.

#### 2.1.4 Spike-based interaction with phase delay

After neural encoding and eccentric down-sampling, the information within the ON/OFF pathways is transmitted to the second, spiking interaction layer, further extracting looming motion features. In the ON pathway, the excitatory input will be directly conveyed to the next summation sub-layer without time delay by convolving surrounding spikes as EPSC. Meanwhile, the inhibitory input is phase-delayed by convolving surrounding delayed spikes as IPSC. The computations in the OFF pathway can be obtained in the same way. The entire process can be defined as


(18) $$\begin{array}{l} E_{on}\left(\tilde{x},\tilde{y},t\right)=\omega \left(t\right)\left(\overset{1}{\underset{i=-1}{\sum }}\overset{1}{\underset{j=-1}{\sum }}{\tilde{S}}_{on}\left(\tilde{x}\;+\;i,\tilde{y}\;+\;j,t\right)W_{1}\left(\tilde{x}\;+\;i,\tilde{y}\;+\;j\right)\right)\rho \end{array}$$

(19) $$\begin{array}{l} I_{on}\left(\tilde{x},\tilde{y},t\right)=\omega \left(t-\epsilon \right)\left(\overset{4}{\underset{i=-4}{\sum }}\overset{4}{\underset{j=-4}{\sum }}{\tilde{S}}_{on}\left(\tilde{x}\;+\;i,\tilde{y}\;+\;j,t-\epsilon \right)W_{2}\left(\tilde{x}\;+\;i,\tilde{y}\;+\;j\right)\right)\rho \end{array}$$

(20) $$\begin{array}{l} E_{off}\left(\tilde{x},\tilde{y},t\right)=\omega \left(t\right)\left(\overset{1}{\underset{i=-1}{\sum }}\overset{1}{\underset{j=-1}{\sum }}{\tilde{S}}_{off}\left(\tilde{x}\;+\;i,\tilde{y}\;+\;j,t\right)W_{1}\left(\tilde{x}\;+\;i,\tilde{y}\;+\;j\right)\right)\rho \end{array}$$

(21) $$\begin{array}{l} I_{off}\left(\tilde{x},\tilde{y},t\right)=\omega \left(t-\epsilon \right)\left(\overset{4}{\underset{i=-4}{\sum }}\overset{4}{\underset{j=-4}{\sum }}{\tilde{S}}_{off}\left(\tilde{x}\;+\;i,\tilde{y}\;+\;j,t-\epsilon \right)W_{2}\left(\tilde{x}\;+\;i,\tilde{y}\;+\;j\right)\right)\rho \end{array}$$

where $E_{on/off}\left(\tilde{x},\tilde{y},t\right)$ represents the EPSC and $I_{on/off}\left(\tilde{x},\tilde{y},t\right)$ denotes the IPSC. ϵ indicates the delayed phase time, and *W*_1_, *W*_2_ are spatial convolution matrices, obeying Gaussian distribution to adjust the connection weights of intermediate neurons. Although the previous ANN methods apply similar strategy to shape the looming selectivity, e.g., Fu et al. (2018, 2020a), the proposed SLoN differs mainly in the following aspects:

- The SLoN is more biologically plausible as transmitting and processing action potentials between connected neurons.
- The looming selectivity can be implemented through spatiotemporal competition between excitation and inhibition, as demonstrated in previous modeling studies (Fu et al., 2019; Fu, 2023). Here, we introduce the new concept of phase delay to reflect the spatiotemporal competition between non-delayed EPSC and delayed IPSC.
- The EPSC is generated by the activities of a relatively smaller area of excitatory input spikes; on the other hand, the IPSC has a larger impact despite being delayed.

Therefore, the competitive interaction between EPSC and IPSC within the ON/OFF channels happened at the summation sub-layer as


(22) $$\begin{array}{l} G_{on}\left(\tilde{x},\tilde{y},t\right)=E_{on}\left(\tilde{x},\tilde{y},t\right)-I_{on}\left(\tilde{x},\tilde{y},t\right) \end{array}$$

(23) $$\begin{array}{l} G_{off}\left(\tilde{x},\tilde{y},t\right)=E_{off}\left(\tilde{x},\tilde{y},t\right)-I_{off}\left(\tilde{x},\tilde{y},t\right) \end{array}$$

The summation LIF neuron receives remaining current injection, causing an increase in neuronal membrane potential. If there is no input spikes, the neuron's membrane potential decays to the resting potential level. On the other hand, when the membrane potential exceeds the threshold, the neuron fires a spike, and the potential immediately declines to the resting level. Such neuronal dynamics can be described as


(24) $$\hat{S_{on}}(\tilde{x},\tilde{y},t)=U(V_{on}(\tilde{x},\tilde{y},t_{3})e^{-\frac{dt}{\tau }}+G_{on}(\tilde{x},\tilde{y},t)-\omega (t)\rho ))$$

(25) $$\begin{array}{l} V_{on}\left(\tilde{x},\tilde{y},t\right)=V_{on}\left(\tilde{x},\tilde{y},t_{3}\right)e^{-\frac{dt}{\tau }}+G_{on}\left(\tilde{x},\tilde{y},t\right)-\omega \left(t\right)\hat{S_{on}}\left(\tilde{x},\tilde{y},t\right)\rho \end{array}$$

(26) $$\hat{S_{off}}(\tilde{x},\tilde{y},t)=U(V_{off}(\tilde{x},\tilde{y},t_{4})e^{-\frac{dt}{\tau }}+G_{off}(\tilde{x},\tilde{y},t)-\omega (t)\rho ))$$

(27) $$\begin{array}{l} V_{off}\left(\tilde{x},\tilde{y},t\right)=V_{off}\left(\tilde{x},\tilde{y},t_{4}\right)e^{-\frac{dt}{\tau }}+G_{off}\left(\tilde{x},\tilde{y},t\right)-\omega \left(t\right)\hat{S_{off}}\left(\tilde{x},\tilde{y},t\right)\rho \end{array}$$

where *U*(·) is the Heaviside step function, and *t*_3_, *t*_4_ indicate the moment when last spike was emitted from corresponding neurons within ON/OFF channels, respectively. $V_{on/off}\left(\tilde{x},\tilde{y},t\right)$ represents the membrane potential, and $\hat{S_{on/off}}\left(\tilde{x},\tilde{y},t\right)$ represents whether the neuron at $\left(\tilde{x},\tilde{y}\right)$ releases a spike at *t* phase time. Notably, the spike weight ω(*t*) and the threshold ρ are associated with the calculation of neuronal membrane potential and spike.

#### 2.1.5 ON/OFF-type LIF neurons and feed-forward inhibition

Subsequently, there are ON/OFF-type LIF neurons, pooling spikes from ON/OFF channels. Notably, the summation sub-layer and LIF neurons are fully connected with a global Gaussian distributed matrix *W*_3_ for either ON/OFF pathways. Inspired by the locust's LGMD computational models (Fu et al., 2019), here we introduce ON/OFF-type feed-forward inhibition (FFI) mechanism which works effectively to suppress directly the ON/OFF-type LIF neurons if large area of RF is highly activated inside a tiny time window. The previous research has demonstrated the effectiveness of FFI mechanism to prohibit the model from responding to the non-collision stimuli including rapid view shifting. Differently to the previous computations of FFI, we nevertheless compute the population firing rate of ON/OFF-type events to determine its efficacy. The computation can be defined as


(28) $$F_{on}(t)=\omega (t-\epsilon )(\frac{\sum_{i=1}^{DL}\sum_{j=1}^{DL}{\tilde{S}}_{on}(\tilde{x},\tilde{y},t-\epsilon )}{DL^{2}})$$

(29) $$F_{off}(t)=\omega (t-\epsilon )(\frac{\sum_{i=1}^{DL}\sum_{j=1}^{D7}{\tilde{S}}_{off}(\tilde{x},\tilde{y},t-\epsilon )}{DL^{2}})\;$$

where *DL* represents the length of down-sampled image after EC. Notably, the phase weight ω is also coupled with the calculation yet with the phase delay ϵ. The FFI output decides whether the ON/OFF-type LIF neurons receive spikes from the ON/OFF channels. Precisely, when the population rate exceeds a threshold, the LIF neuron will be directly suppressed, not receiving any input spikes. The computation can be expressed as


(30) $${\tilde{I}}_{on}(t)=\{\begin{array}{ll} \omega (t)(\sum_{i=1}^{DL}\sum_{j=1}^{DL}\hat{S_{on}}\;(i,j,t)W_{3}(i,j))\rho & \text{ if }F_{on}(t)<S_{th}\\ 0 & \text{ if }F_{on}(t)\geq S_{th} \end{array}$$

(31) $${\tilde{I}}_{off}(t)=\{\begin{array}{ll} \omega (t)(\sum_{i=1}^{DL}\sum_{j=1}^{DL}\hat{S_{off}}\;(i,j,t)W_{3}(i,j))\rho & \text{ if }F_{off}(t)<S_{th}\\ 0 & \text{ if }F_{off}(t)\geq S_{th} \end{array}$$

where *S*_*th*_ is the threshold. Otherwise, the ON/OFF-type LIF neurons are charged by input spikes working as the following dynamics:


(32) $$S_{on}^{-}(t)=U({\tilde{V}}_{on}(t_{5})e^{-\frac{dt}{\tau }}+{\tilde{I}}_{on}(t)-\omega (t)\rho ))$$

(33) $$S_{off}^{-}(t)=U({\tilde{V}}_{off}(t_{6})e^{-\frac{dt}{\tau }}+{\tilde{I}}_{off}(t)-\omega (t)\rho ))$$

(34) $$\begin{array}{l} {\tilde{V}}_{on}\left(t\right)={\tilde{V}}_{on}\left(t_{5}\right)e^{-\frac{dt}{\tau }}+{\tilde{I}}_{on}\left(t\right)-\omega \left(t\right)\bar{S_{on}}\left(t\right)\rho \end{array}$$

(35) $$\begin{array}{l} {\tilde{V}}_{off}\left(t\right)={\tilde{V}}_{off}\left(t_{6}\right)e^{-\frac{dt}{\tau }}+{\tilde{I}}_{off}\left(t\right)-\omega \left(t\right)\bar{S_{off}}\left(t\right)\rho \end{array}$$

Similarly, *t*_5_, *t*_6_ indicate the moment of last emitted spike from ON/OFF-type LIF neurons, respectively. $\bar{S_{on/off}}\left(t\right)$ represents the generated spike in current phase time, and Ṽ_*on*/*off*_(*t*) is the neuronal membrane potential.

#### 2.1.6 SLoN output neuron

As shown in Figure 1, the SLoN has only one output neuron to indicate whether there is any potential looming motion identified by the network. The outputs of ON/OFF-type neurons are linearly combined at the SLoN output neuron, which is


(36) $$\begin{array}{l} I\left(t\right)=\omega \left(t\right)\left(\theta_{1}\bar{S_{on}}\left(t\right)+\theta_{2}\bar{S_{off}}\left(t\right)\right) \end{array}$$

where {θ_1_, θ_2_} are term coefficients to adjust the different selectivity to ON/OFF-contrast looming stimuli. We will demonstrate the selection of such parameters in the experiments. Finally, the output of the SLoN is calculated as


(37) $$S(t)=U(V(t_{7})e^{-\frac{dt}{\tau }}+I(t)-\omega (t)\rho ))$$

(38) $$\begin{array}{l} V\left(t\right)=V\left(t_{7}\right)e^{-\frac{dt}{\tau }}+I\left(t\right)-\omega \left(t\right)S\left(t\right)\rho \end{array}$$

where *U*(·) is the Heaviside step function, *t*_7_ indicates the moment of last emitted spike, and *S*(*t*), *V*(*t*) represent the spike and the membrane potential of the SLoN output neuron, respectively. The detailed online signal processing algorithms are also shown in Algorithm 1.

**Algorithm 1:**
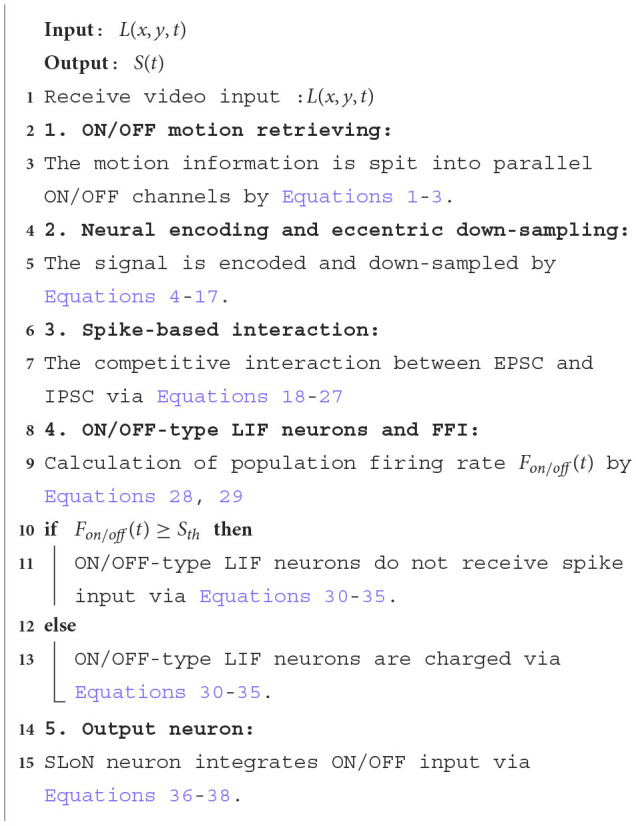
Online algorithm of the SLoN.

### 2.2 Setting the parameters

The parameters and their settings are shown in Table 2. It is essential to emphasize that the SLoN processes visual signals in a feed-forward structure without any feedback connections. Furthermore, learning methods were not employed in this modeling endeavor. The primary focus of this study was to propose an SNN framework that emulates biological visual neural systems for looming perception and integrates ON/OFF channels for eccentric neural encoding. The parameters were determined with considerations of mainly two aspects as follows:

- Certain parameters, such as the LIF spiking threshold and the standard deviations in connection matrices, were adjusted during experimental validation to enhance looming perception performance. The paramount consideration was achieving robust looming selectivity tested across diverse visual scenarios.
- On the other hand, the SLoN drew modeling inspiration from several notable studies: (1) The parameters of PC with weighted spikes were adapted from the modeling study by Kim et al. (2018), and the novel concept of PD was further investigated in the experiments. (2) The parameters of EC were derived from the modeling study by D'Angelo et al. (2020), and we provided a more detailed description of the EC algorithms. (3) The fundamental structure of ON/OFF channels was based on recent biological and computational advancements in visual motion perception (Fu, 2023).

**Table 2:** Parameters of the SLoN.

**Parameter**	**Description**	**Value**
α_1_	Coefficient at signal bifurcation of the neural encoding layer (Equations 2, 3)	0.1
*max*(*R*^*s*^)	Largest size of RF in Equation 8	10
τ	Time constant in Equations 9, 16, 24, 25, 26, 27, 32–35, 37, 38	$\frac{1,000}{fps}$
*R*_*k*_	Percentage area of the RF in Equation 9	0.6
ρ	LIF neuron firing threshold in Equations 10, 17–21, 24–27, 30–35, 37, 38	0.9
σ_1_	Standard deviation of Gaussian kernel in Equations 18, 20	1
σ_2_	Standard deviation of Gaussian kernel in Equations 19, 21	0.5
σ_3_	Standard deviation of Gaussian kernel in Equations 30, 31	1
ϵ	Phase delay time in Equations 19, 21, 28, 29	2
L	Side length of original input visual stimuli	100
DL	Down-sampled side length in Equations 28–31	58
*S*_*th*_	Threshold of FFI population rate in Equations 30, 31	0.1
{θ_1_, θ_2_}	Term coefficients in Equation 36	{0.5, 0.5}

### 2.3 Setting the experiments

To validate our proposed SLoN and investigate its key components, we conducted experiments across four categories, encompassing computer-simulated and real-physical stimuli. The goals were to demonstrate: (1) the basic functionality of the proposed model for looming perception with robust selectivity, (2) the efficacy of the ON/OFF channels in implementing diverse selectivity to ON/OFF-contrast looming stimuli, (3) the impact of the inaugurated concept of phase delay on looming perception, and (4) the results of an ablation study with comparison on down-sampling methods in the SLoN model.

Specifically, the experimental videos were divided into two parts. The first part included computer-simulated movements such as approaching, receding, translating, and grating stimuli, aligning with related physiological research. The second part involved more challenging real-world vehicle crash videos from a dashboard camera recording, which was sourced from related modeling research (Fu et al., 2020b). All input videos were of size 100 × 100 pixels, sampled at 30Hz. The source code of SLoN and visual stimuli used in the experiments can be accessed as open source at Github.

## 3 Results and analysis

Within this section, we present the results of systematic experiments with analysis. This includes four categories of experiments to verify the effectiveness of the proposed SLoN model. The main objectives of these tests fall within the following scope of

- Verifying the effectiveness of SLoN for looming perception against a variety of visual collision challenges
- Implementing the different looming selectivity to ON/OFF-contrast aligned with biological principles
- Demonstrating the efficacy of phase delay in spike-based interaction shaping looming selectivity
- Highlighting the robustness of EC mechanism increasing the fidelity of looming perception across various complicated scenarios.

### 3.1 Basic functionality

**Figure 4:**
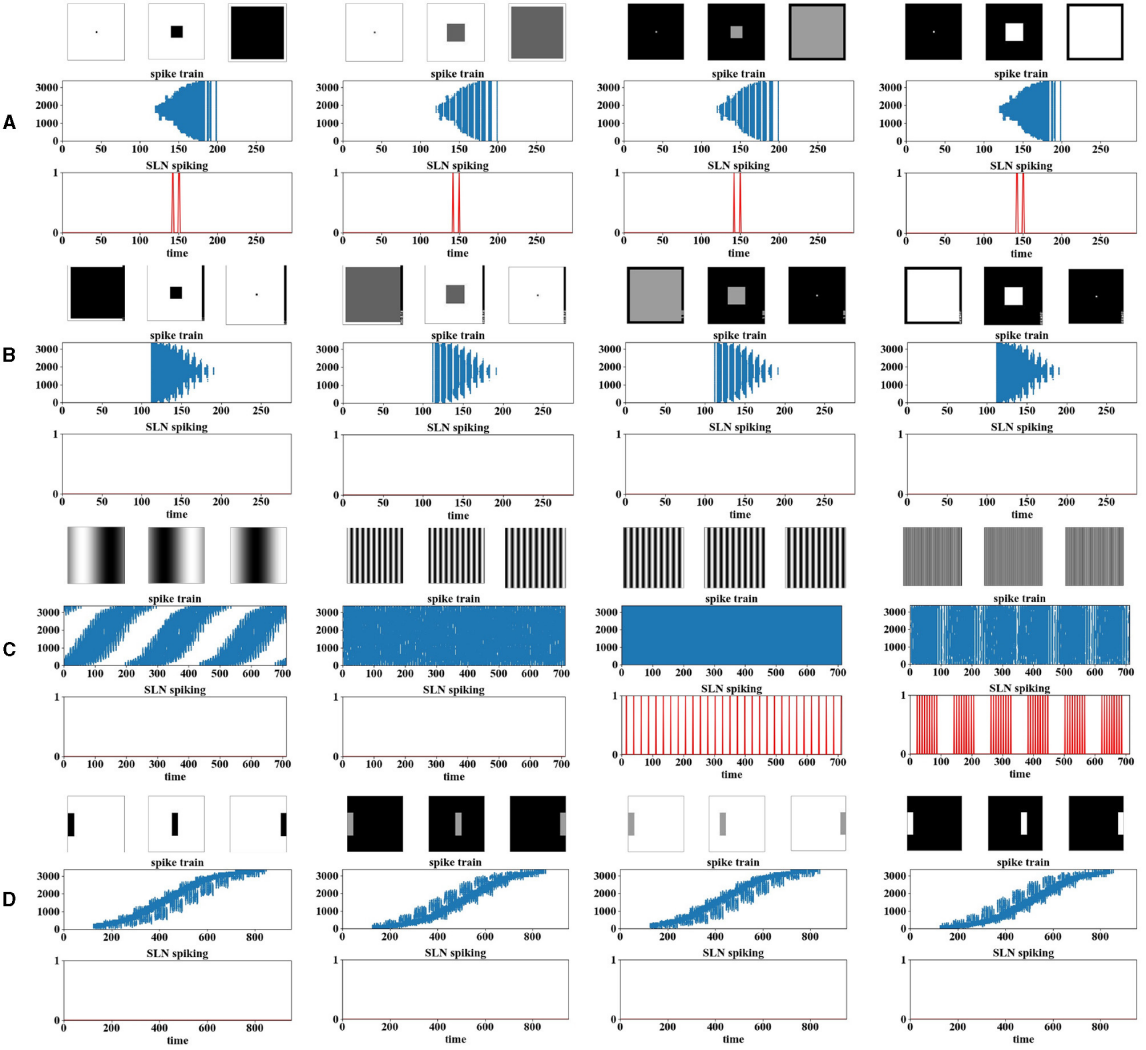
The results of the computer-simulated movements are presented, including **(A)** looming, **(B)** receding, **(C)** grating, and **(D)** translating. The images above the experimental results are snapshots of the input stimuli. The results are organized into two sub-graphs each, with one illustrating the position of the activated neurons over time as raster plots (i.e., the spike trains), and the other depicting the final spike output of the model. In the spike trains, the *X*-axis represents the phase time, while the *Y*-axis indicates ON/OFF-events in a vector after phase coding. In the model response, the *X*-axis indicates the phase time, and the *Y*-axis indicates the binary output of spikes.

For collision perception models, the most important property to achieve is whether the model can respond most strongly to moving object signaling approaching rather than other types of movements. This is also naturally one criterion of “looming selectivity” for computer models. First, we carried out experiments on the approaching of squares, at four contrasts, as shown in Figure 4A. We can observe that the SLoN spikes for either white or dark objects, and the contrast matters. The encoded spike trains show more denser events within ON/OFF channels delivered to the spiking interaction layer, and the events of action potentials spread out spatially with respect to the approaching of squares. At all the tested contrasts, the SLoN performs stably on perception of looming motion.

On the other hand, we also did experiments with objects receding to observe if the model would generate spike to this opposite stimulus. Figure 4B shows, conversely, the spatiotemporal distributions of spike trains challenged by receding stimuli. Interestingly, the SLoN does not generate any spikes in this case, marking the emergence of looming selectivity.

Additionally, we tested the SLoN with stimuli involving translating movements, including sinusoidal grating at different frequencies and single-bar crossing at four contrasts. Figure 4C demonstrates that the SLoN does not consistently respond to grating at certain frequencies but is regularly activated in some cases. We observed that the PD and the threshold of FFI population rate *S*_*th*_ can influence the SLoN's response to grating. Increasing the threshold could suppress the SLoN's response to grating stimuli but might impact the perception of looming motion if set too high. The subsequent experiments in Figure 8 will illustrate the influence of PD on grating stimuli. While adjusting the delay can suppress the response to grating stimuli, it can also compromise the model's ability to detect collisions. Attempts to make the model resolve both grating and approaching stimuli by adjusting the activation threshold within the neuron model were not entirely successful in distinguishing between approaching and receding stimuli. Moreover, the proposed EC facilitates the SLoN model in capturing motion features near the center of view. Zooming in on the grating pattern reveals motion features that were not discernible before the implementation of EC. Raising the threshold could suppress the SLoN's response to grating stimuli but may affect the perception of looming motion. Additionally, the spike trains, as shown in Figure 4D, display action potential events as the bar moves rightward across the RF, and the SLoN remains inactive against such motion patterns.

**Figure 5:**
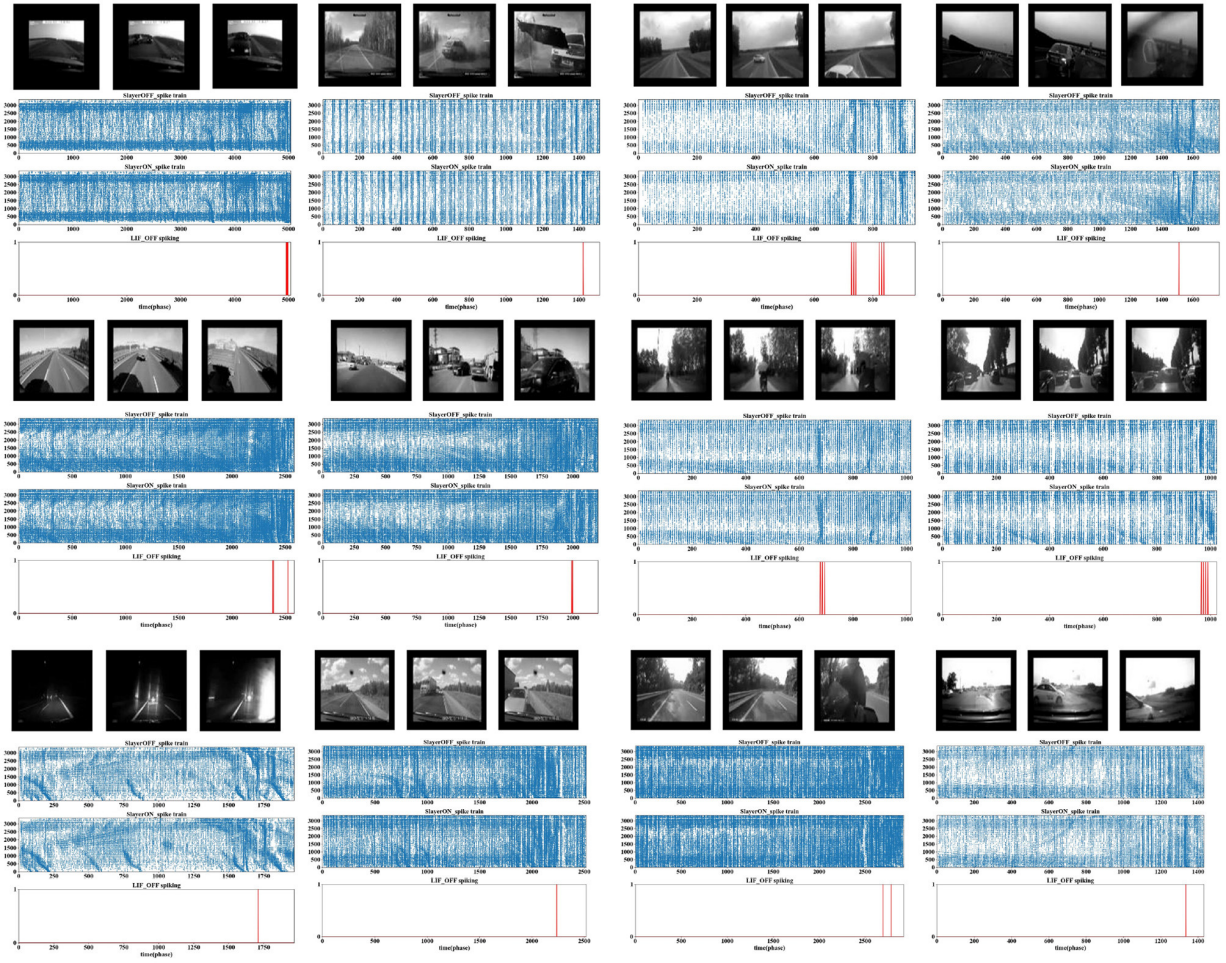
The results of vehicle crash scenarios are depicted, with the images above the experimental results illustrating each stage of collision events. The results are presented in three sub-graphs: the top panel represents the position of the S cells activated in the OFF channel, the middle panel represents the position of the S cells activated in the ON channel, and the bottom panel represents the final spike output of the model. The *Y*-axis indicates binary pulse events, and the *X*-axis elaborates on phase time. The SLoN releases spikes for collision perception right before the colliding moment while remaining quiet in normal navigation, as challenged by 12 highly complex scenes.

To increase the challenge, we conducted experiments using real-world car crashes recorded by dashboard cameras. The SLoN model exhibits robust performance against various crash scenarios, generating brief spikes just before the moment of collision (Figure 5). Due to the high complexity of the vehicle scenes and the redundancy of spike trains after down-sampling, we present the activation location of ON/OFF polarity S-cells instead of EC cells. It could be difficult to find collision events directly from such spike train patterns. Nevertheless, the proposed SLoN model works effectively to filter out irrelevant information, accurately extracting looming motion cues.

### 3.2 Potential of ON/OFF channels

The preceding bio-inspired collision perception visual systems demonstrate the capability to achieve diverse looming selectivity to ON/OFF-contrast, representing darker or brighter objects moving against a background. Specifically, the neural network model proposed by Fu et al. (2018) achieves the selectivity of the locust's LGMD1 neuron, responding to both dark and white looming objects. With the introduction of biased-ON/OFF channels, the neural model presented by Fu et al. (2020a) realizes the unique selectivity of the locust's LGMD2 neuron to only darker looming objects. In a recent study (Chang et al., 2023), feedback neural computation is incorporated into the original model based on ON/OFF channels to achieve diverse selectivity to either ON/OFF-contrast looming stimuli.

**Figure 6:**
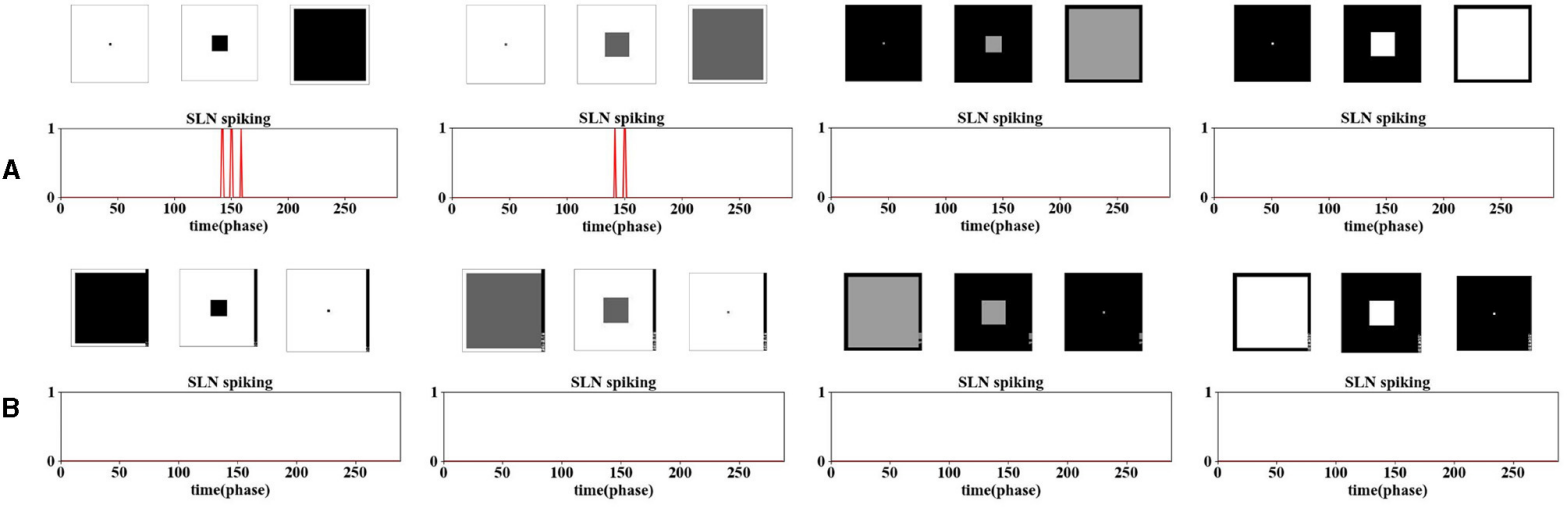
The illustration of ON/OFF channels in SLoN showcases the specific selectivity to only darker approaching objects rather than other categories of movements. **(A)** presents results of two darker and two brighter objects approaching. **(B)** presents results of four contrasting objects receding from the RF.

To align with the functionality of ON/OFF channels revealed in neuroscience (Fu, 2023), we conducted experiments to achieve a similar selectivity to the aforementioned LGMD2 neuron model, which is only sensitive to darker approaching objects, signaling OFF-contrast. The experiments so far have demonstrated that the SLoN model can achieve the selectivity of the locust's LGMD1, responding to the approaching of both white and dark objects. Figure 6 illustrates situations involving white and dark objects approaching and receding. As our proposed SLoN combines ON/OFF-type LIF neurons at the final output layer, we have the flexibility to adjust the contribution of either channel to the final LIF neuron. In previous experiments, ON-type and OFF-type spikes contributed equally. However, in these specific tests, we adjusted the coefficients in Equation 36, with the coefficient for the OFF pathway increased to 0.7 and that of the ON pathway decreased to 0.3. This adjustment indicates that OFF-type spikes contribute relatively more to the network. Consequently, as shown in Figure 6, the SLoN only generates spikes for dark looming patterns against a white background, at two contrasts, over any other movements. The model also exhibits a higher frequency of spikes in response to darker looming stimuli.

It is noteworthy that, unlike the model of the locust's LGMD2 proposed in the study by Fu et al. (2020a), which also responds briefly to a white object receding within a dark background (OFF-contrast), the proposed SLoN can better distinguish approaching from receding courses. Therefore, the ON/OFF channels in the SLoN contribute potentially to enriching the selectivity to align with the requirements of looming detection in more complex, real-world scenarios.

### 3.3 Investigation of phase delay

The phase time delay is a novel concept introduced in this modeling work. This subsection delves into the selection of the delay window in looming perception. As shown in Section 3.1, the delay is a crucial parameter that empowers our model to address the grating problem and refine the looming selectivity. The default setting for the delay is two phases, as shown in Table 2. In this investigation, we explored a range between 0 and 8, where 0 implies no delay in the IPSC, while 8 indicates that the IPSC is delayed to the same phase as the previous video frame.

**Figure 7:**
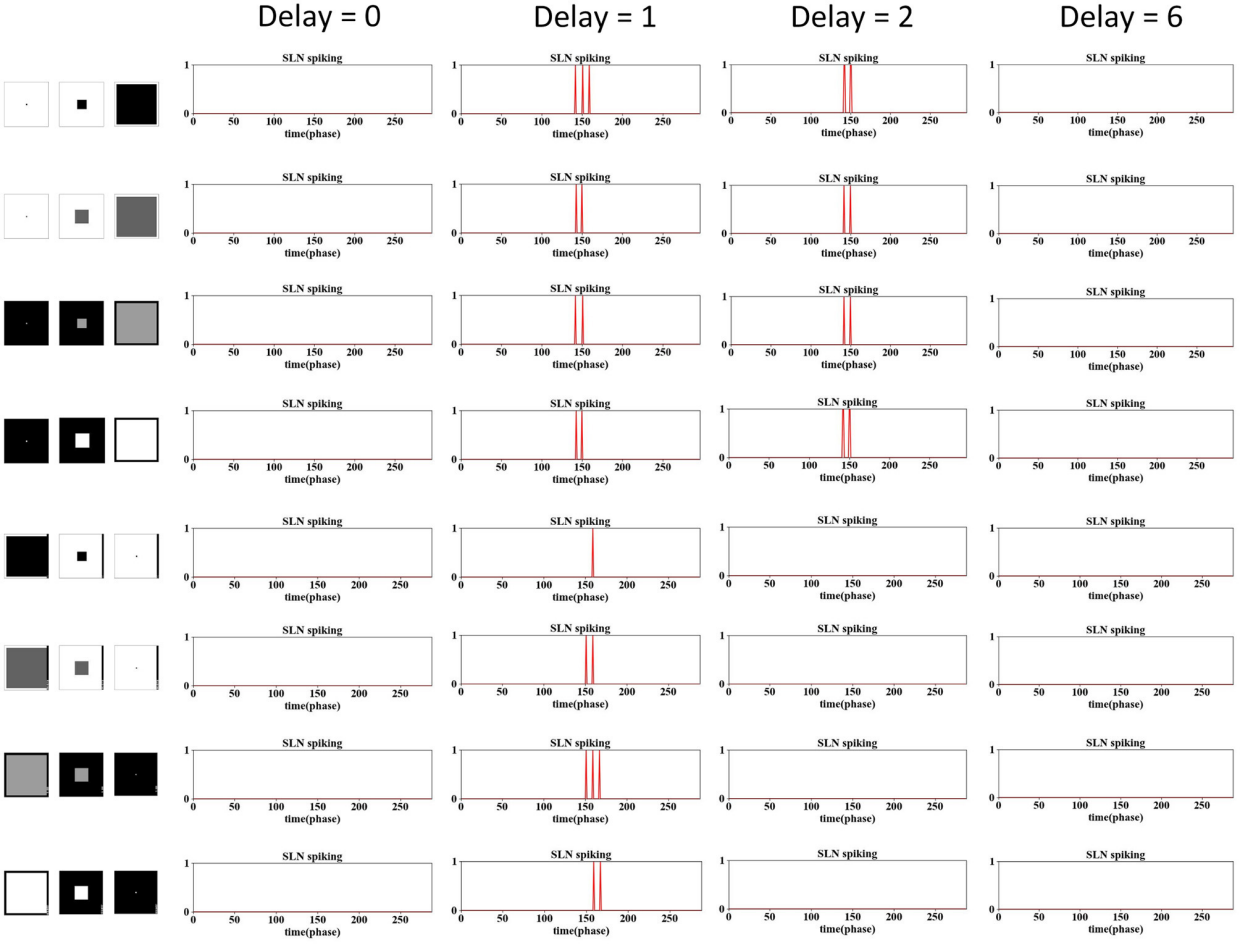
The examination of phase delay in the SLoN concerning approaching and receding stimuli is conducted using computer-simulated movements, including dark/white approaching and receding scenarios. The SLoN model with a phase delay of ϵ = 2 proves to be optimal, aligning with expectations, by responding exclusively to approaching stimuli.

**Figure 8:**
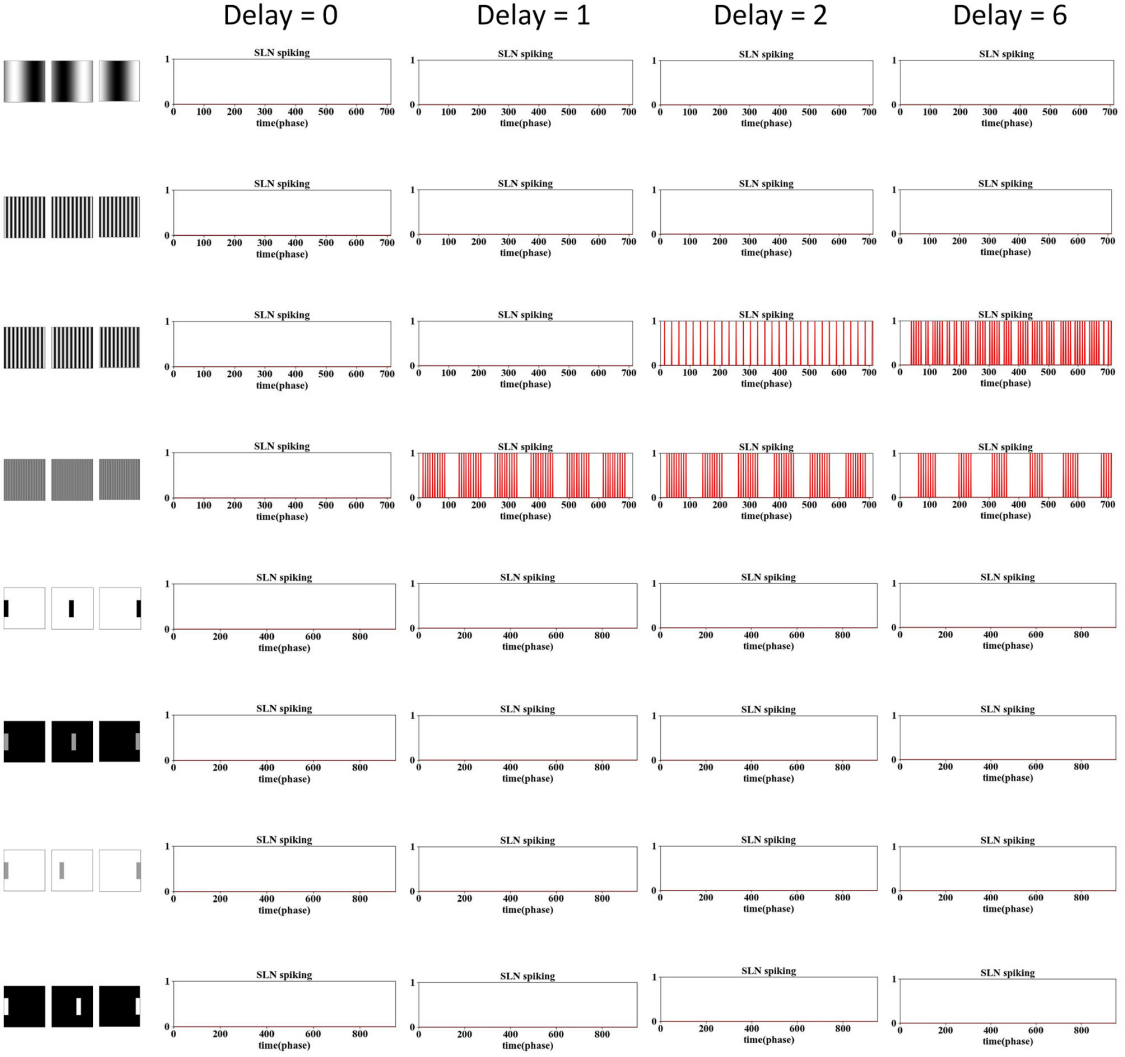
The examination of phase delay in the SLoN concerning grating and translating stimuli is conducted using computer-simulated movements. Four different frequencies of grating movements are tested. The SLoN model with a phase delay of ϵ = 0 outperforms other comparative tests in this case, indicating that the SLoN should not respond to any grating or translating stimuli.

The results are shown in Figures 7, 8, encompassing various motion patterns, with only representative results showcased. When the delay is set to 0, the SLoN model does not respond to any stimulus, including dark/white approaching, receding, translating, and grating movements, rendering it devoid of motion sensitivity. This underscores the crucial role of phase delay in constructing a motion perception SNN when utilizing phase coding to transform motion features into ON/OFF-type spike trains. The spatiotemporal competition between excitation and inhibition consistently proves to be pivotal in shaping the specific looming selectivity as affirmed by numerous prior studies (Fu, 2023).

Obviously, when the PD increases to 1, the SLoN works effectively to detect approaching stimuli, however, also responds to receding stimuli. After further increasing the PD to 2, the SLoN can distinguish between approach, receding very well which is more in line with the expected looming selectivity. A limitation of the SLoN model is its inability to effectively handle grating movements, as it continues to be activated by certain frequencies, resulting in regular clustering of spikes. Addressing this issue requires the introduction of new bio-plausible mechanisms and algorithms in future research. Additionally, the PD cannot be set too large, as shown in Figures 7, 8, since the SLoN loses its ability for looming perception. We further investigated real-world collision challenges with different delays, and the results are shown in Table 3.

**Table 3:** Results of different phase delay in the SLoN against real-world collision challenges.

**Dataset**	**Delay 0**	**Delay 1**	**Delay 2**	**Delay 3**	**Delay 4**	**Delay 5**	**Delay 6**	**Delay 7**	**Delay 8**
Car collision1	✕	✓	✓	✓	✓	✓	✓	✓	✕
Car collision2	✕	✕	✓	✓	✕	✓	✕	✓	✕
Car collision3	✕	✓	✓	✓	✓	✓	✓	✓	✕
Car collision4	✕	✓	✓	✓	✕	✓	✕	✓	✕
Car collision5	✕	✓	✓	✓	✕	✓	✓	✓	✕
Car collision6	✕	✓	✓	✓	✕	✓	✓	✓	✕
Car collision7	✕	✓	✓	✓	✕	✓	✕	✓	✕
Car collision8	✕	✓	✓	✓	✓	✓	✓	✓	✕
Car collision9	✕	✕	✓	✕	✕	✕	✓	✕	✕
Car collision10	✕	✓	✓	✓	✕	✓	✓	✓	✕
Car collision11	✕	✕	✓	✓	✕	✓	✓	✕	✕
Car collision12	✕	✕	✓	✕	✕	✕	✕	✕	✕
Ball collision1	✕	✓	✓	✓	✕	✓	✓	✓	✕
Ball collision2	✕	✕	✓	✕	✕	✕	✕	✕	✕
Ball collision3	✕	✕	✓	✕	✕	✕	✕	✕	✕
Ball collision4	✕	✕	✓	✕	✕	✕	✕	✕	✕

The notation “delayX” indicates that X-phases are delayed in the SLoN model.

✕ indicates the model fails to looming perception while ✓indicates true positives.

In summary, our investigation indicates that a PD of two-phases is an optimal temporal delay for the interaction between EPSC and IPSC in the network. This choice corresponds to the concentration of ON/OFF-type spike events, which are more heavily weighted within the initial phases through phase coding. This observation suggests that the most pertinent information is effectively conveyed to the ON/OFF-type LIF neurons during the early time window of each video frame. However, the current analyses are solely based on experiments where the mathematical analysis of PD should be involved in the future work.

### 3.4 Ablation study on eccentric down-sampling

Photoreceptors density in the mammalian retina is high at the fovea and decreases toward the periphery area. A recent study (D'Angelo et al., 2020) combined event-driven visual processing to model sEMD with the non-uniform retina model as a down-sampling of the visual field. We agreed with this strategy mimicking mammalian motion vision and incorporated the EC mechanism in the neural encoding of ON/OFF channels. Other motivations for us to model EC were (1) further sharpening up the looming selectivity as the object normally expands from the center area of visual field and (2) enhancing the robustness across complex visual scenes as the EC could potentially reduce noisy optical flows resident in the periphery. Accordingly, this subsection carries on ablation study on the down-sampling method and compares the EC with typical uniform down-sampling in the framework by SLoN's.

**Figure 9:**
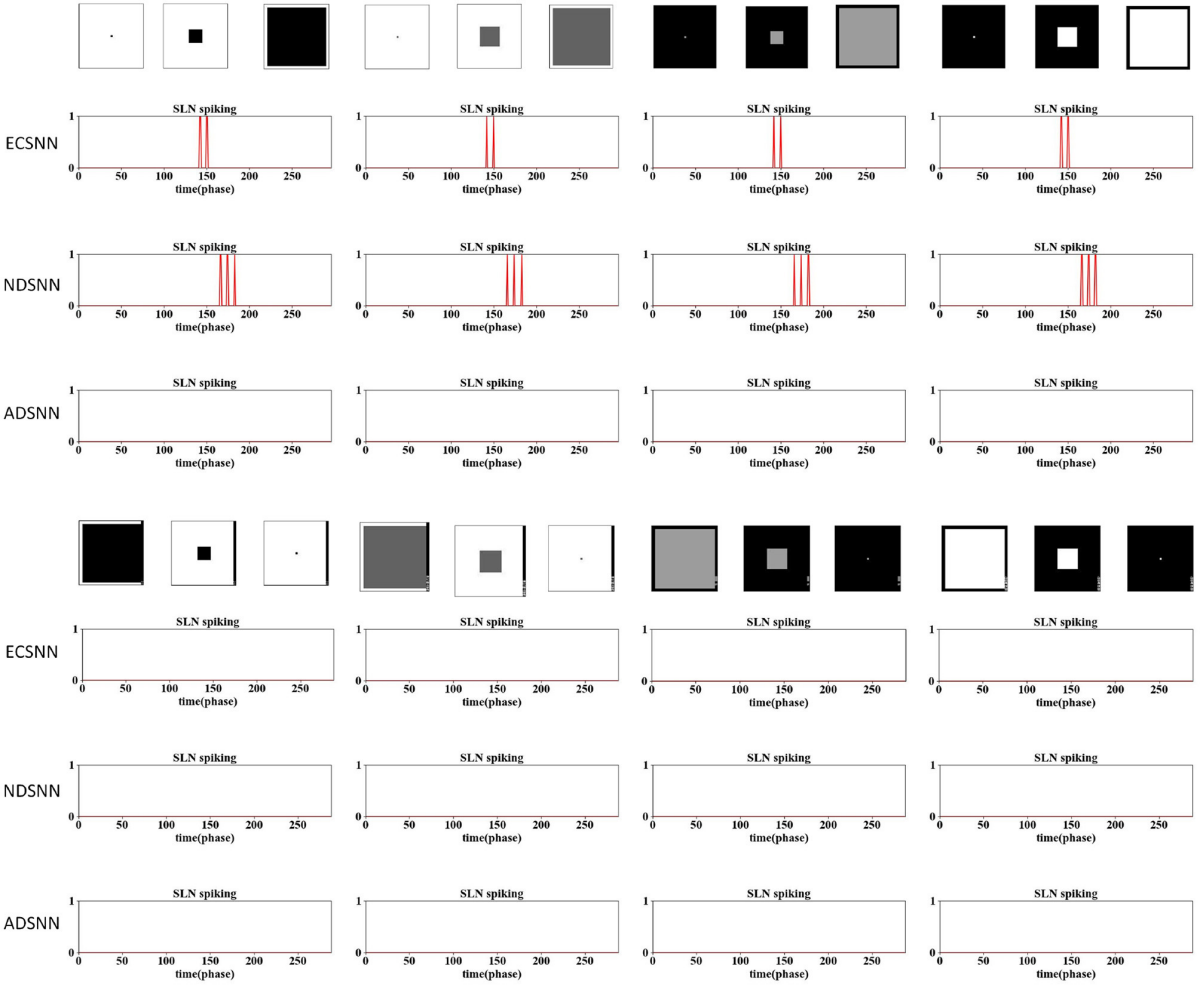
In the comparative results of the ablation study on down-sampling methods against dark/white looming and receding stimuli, ECSNN represents the SLoN with EC down-sampling method, NDSNN represents the SLoN without any down-sampling method, and ADSNN represents the SLoN with the traditional average down-sampling method. Both ECSNN and NDSNN can distinguish between approaching and receding movements, with NDSNN demonstrating a higher spiking rate. However, ADSNN cannot respond to looming stimuli.

**Figure 10:**
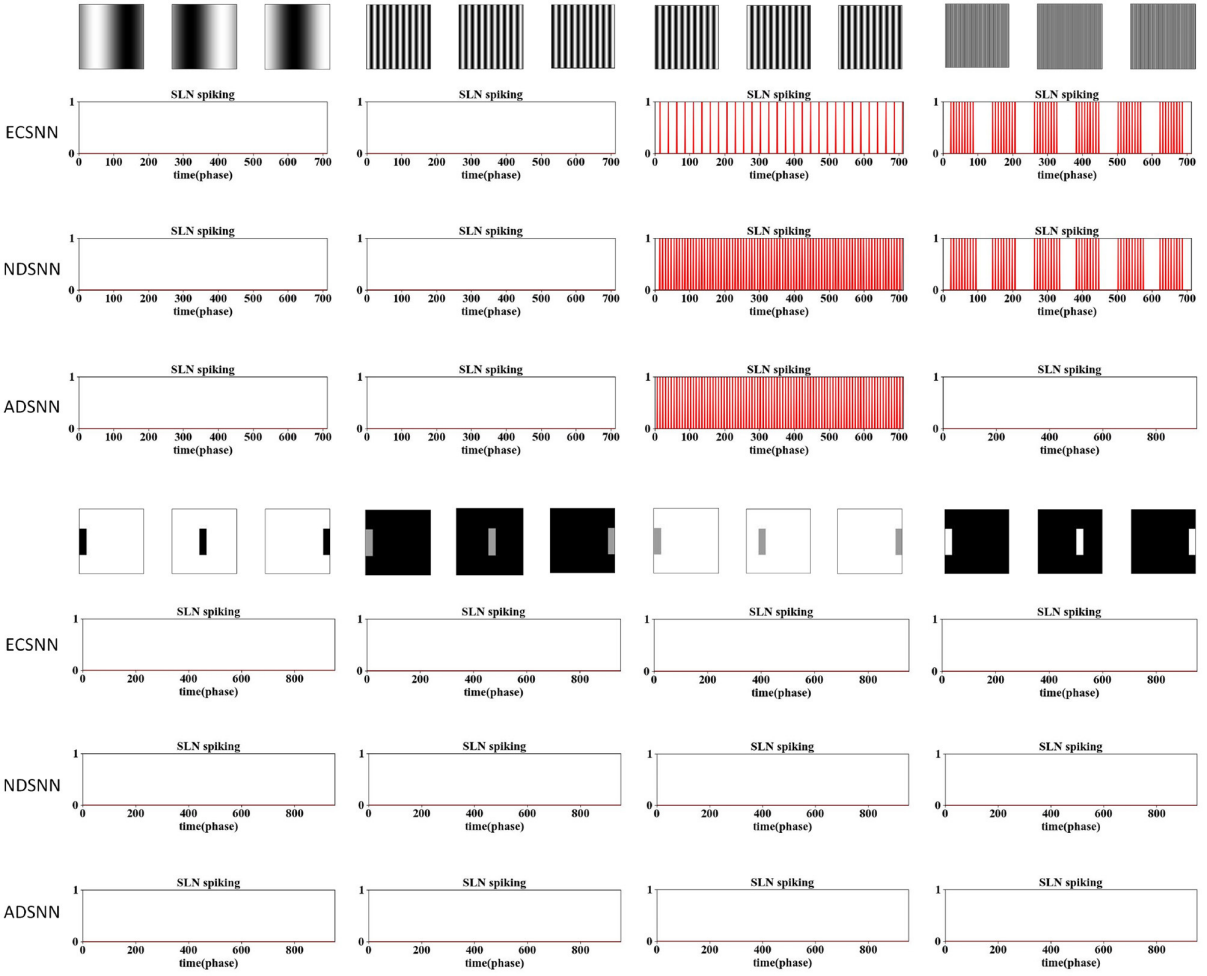
Comparative results of ablation study on down-sampling methods against grating and dark/white translating stimuli following Figure 9.

**Figure 11:**
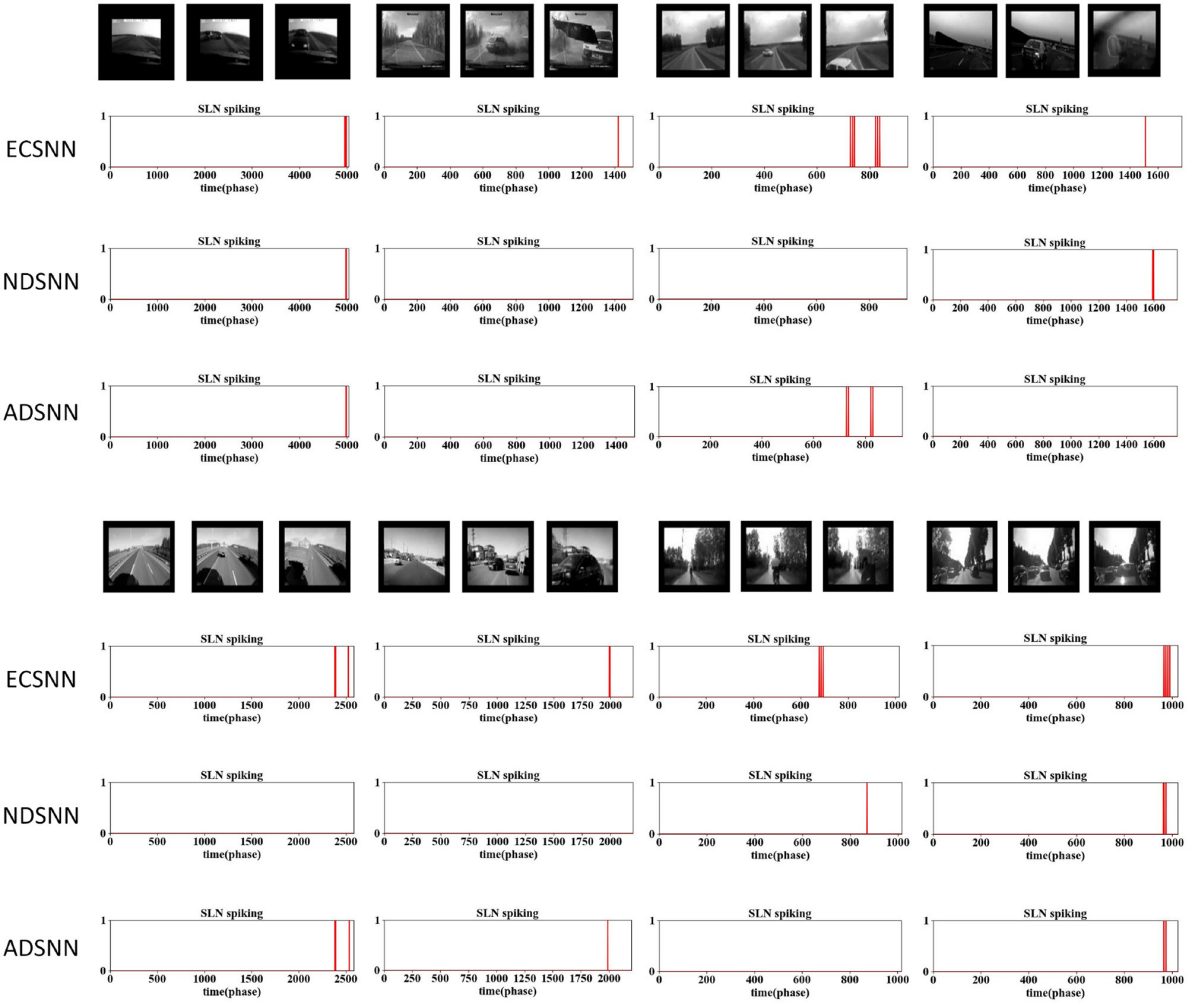
Comparative results of the ablation study on down-sampling methods against complex vehicle crash scenarios: the proposed SLoN with EC works most robustly to detect all imminent collisions in real-world scenes.

**Figure 12:**
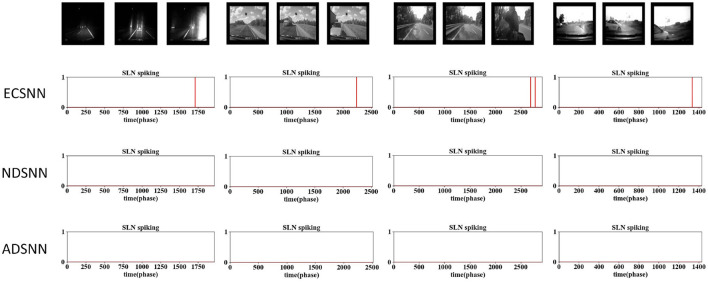
Comparative results of the ablation study on down-sampling methods against complex vehicle crash scenarios: the proposed SLoN with EC outperforms other models in real-world scenes.

The results are shown in Figures 9–12. Three sets of experiments were conducted for each investigated model, including the EC down-sampling SLoN (ECSNN), the SNN with no down-sampling (NDSNN), and the average down-sampling SLoN (ADSNN). The synthetic stimuli, as shown in Figures 9, 10, revealed similar behavior between ECSNN and NDSNN. Both models responded to white/dark objects approaching but not to objects receding and translating. However, they were not effective in addressing the grating problem at certain frequencies.

Furthermore, ECSNN exhibits earlier spikes than NDSNN in response to approaching objects (Figure 9), indicating that the EC mechanism concentrates motion-induced excitations in the central RF. Additionally, NDSNN is more responsive to grating stimuli than ECSNN. Conversely, ADSNN proves ineffective at detecting looming objects and other types of motion stimuli. This study underscores the unsuitability of uniform down-sampling for the proposed algorithm.

To assess the capability of EC down-sampling in improving the model's performance in real-world complex scenarios, experiments with vehicle collision stimuli were conducted. Twelve different scenarios were used to test the three comparative models. The results presented in Figures 11, 12 demonstrate the superior performance of ECSNN compared with the other two models, exhibiting robustness against all collision perception scenarios. Specifically, ECSNN recognizes all collisions, NDSNN detects only four collision events, and ADSNN detects five events. The small size of the RF in the EC-based SLoN model in the foveal region and its larger size in the edge region contribute to its heightened sensitivity to motion at the fovea, aligning with mammalian vision characteristics. The EC mechanism significantly enhances the SLoN model's response to approaching objects. The summarized results of real-world collisions are presented in Table 4, and a couple of additional experiments on ball collisions were included.

**Table 4:** Results of the ablations study on down-sampling methods in the SLoN model against real-world collision challenges.

**Dataset**	**ECSNN**	**ADSNN**	**NDSNN**
Car collision1	✓	✓	✓
Car collision2	✓	✕	✕
Car collision3	✓	✓	✕
Car collision4	✓	✕	✓
Car collision5	✓	✓	✕
Car collision6	✓	✓	✕
Car collision7	✓	✕	✓
Car collision8	✓	✓	✓
Car collision9	✓	✕	✕
Car collision10	✓	✕	✕
Car collision11	✓	✕	✕
Car collision12	✓	✕	✕
Ball collision1	✓	✓	✓
Ball collision2	✓	✕	✕
Ball collision3	✓	✓	✕
Ball collision4	✓	✕	✕

✕ indicates the model fails to looming perception while ✓indicates true positives.

## 4 Discussion

In this study, we introduce a spiking looming perception network (SLoN) inspired by biological motion vision. Our systematic experiments demonstrate the efficacy of the SLoN model in detecting approaching objects while remaining unresponsive to receding and translating ones. Additionally, we showcase the model's robustness in addressing complex challenges posed by real-world scenarios. The investigations into the intrinsic structures of SLoN, encompassing the ON/OFF channels, eccentric down-sampling mechanism, and phase delay, affirm the following key achievements in this modeling research:

- The incorporation of ON/OFF channels in motion-sensitive spiking neural networks enables the implementation of diverse looming selectivity to ON/OFF-contrast stimuli. This capability enhances the stability of model performance in complex and dynamic scenes.
- The newly introduced concept of phase delay extends the theory of phase coding, aligning well with the spatiotemporal interaction between excitatory and inhibitory signals to establish specific looming selectivity. As a result, the proposed SLoN distinguishes itself from typical SNN models that are designed for pattern recognition or object detection.
- The eccentric down-sampling mechanism not only mimics mammalian vision but also aligns with the characteristic of looming perception, focusing more on the expanding edges of objects from the foveal region of the visual field. This feature proves particularly beneficial in complex visual scenes, reducing the model's sensitivity to strong optical flows at the periphery.

Moreover, the SLoN model can achieve the selectivity of locust's LGMD2 (Simmons and Rind, 1997). By adjusting the weighted ON/OFF-type LIF neurons, the OFF channels contribute more than ON channels to the final output neuron. Consequently, the SLoN model achieves specific selectivity for darker objects approaching.

The final ablation experiment also verified the effectiveness of EC down-sampling. The SLoN responds to objects approaching rather than receding and translating. Such fidelity in looming perception is maintained in complex vehicle crash scenarios, which would be practical for utilization in mobile machines. This non-uniform sampling implies that the peripheral RF demands more stimulation to emit spikes. Essentially, the EC mechanism also reduces the dimension of input to the subsequent network with a decreased number of intermediate neurons.

We also note some shortcomings of the SLoN model. First, it cannot address cases of low-contrast motion, i.e., when there is a small luminance difference between the moving target and the background. In such instances, the SLoN model may struggle to detect approaching objects, as the LIF neurons in the initial encoding layer rely on visual contrast for spiking. Additionally, the SLoN model responds to grating movements at certain frequencies, which is not in line with expectations from either biological looming perception circuits or their computational models. Second, the feed-forward inhibition mechanism, learned from the modeling of locust's LGMD neuronal models, works effectively to suppress such stimuli, but to some extent, it influences the responsive preference to approaching stimuli. There is a trade-off in tuning the FFI mechanism, as the threshold is not adaptive at present. Finally, it is essential to note that the SLoN model, like other vision-based neural models, is highly dependent on realistic lighting conditions. Under extreme conditions, such as excessively bright or dark scenes, our model may face challenges.

The future direction of this study encompasses three key aspects. First, we aim to incorporate event-based sensing modalities into the SLoN, providing an ideal match at the neural encoding layer. This approach not only enhances energy efficiency but also offers a high dynamic range, making it adaptable to extreme lighting environments (Gallego et al., 2022). In the SLoN model, the sensor could potentially replace the neural coding layer, directly generating ON/OFF-type spike trains as input for the EC down-sampling, as demonstrated in previous studies (D'Angelo et al., 2020).

Second, in the investigation of phase delay, we have set the maximum value at 8, considering that one video frame is composed of eight phases. However, further research is needed to explore the optimal choice of delay for different types of stimuli. In a previous LGMD2 modeling study (Fu et al., 2020a), inhibitory currents were found to be related not only to the excitatory current of the current frame but also to the excitatory current of the previous frame. Adjusting the delay can partially address specific types of stimuli. The exploration of different delay scopes and whether smaller delays are preferable requires systematic investigation, representing a focus for future work.

Third, our future research will delve into learning methods or time-varying, adaptive mechanisms to effectively handle grating stimuli. This represents a significant area for improvement in the SLoN model.

In conclusion, this study presents a computational model of a spiking looming perception network (SLoN) inspired by biological vision. Our objective is to capture looming perception in a manner consistent with neural information processing in the brain. The proposed model features an eccentric down-sampling mechanism that connects ON/OFF channels for neural encoding and transmission. Notably, we employ phase coding to transform video signals into spike trains for input to the SLoN, enabling the model to process data from frame-based cameras. Additionally, we introduce phase delay to represent the spatiotemporal interaction between excitation and inhibition for achieving looming selectivity. Systematic experiments validate the effectiveness and robustness of the SLoN model across a variety of collision challenges. The foundational structure of SLoN positions it well for integration with event-driven cameras, a focus for future investigations.

## Data availability statement

The original contributions presented in the study are included in the article/supplementary material, further inquiries can be directed to the corresponding author.

## Author contributions

ZD: Data curation, Investigation, Software, Visualization, Writing—original draft. QF: Conceptualization, Methodology, Project administration, Supervision, Validation, Writing—original draft, Writing—review & editing. JP: Formal analysis, Funding acquisition, Supervision, Validation, Writing—review & editing. HL: Formal analysis, Funding acquisition, Supervision, Validation, Writing—review & editing.
